# Combined Effects of Androgen and Growth Hormone on Osteoblast Marker Expression in Mouse C2C12 and MC3T3-E1 Cells Induced by Bone Morphogenetic Protein

**DOI:** 10.3390/jcm6010006

**Published:** 2017-01-05

**Authors:** Kosuke Kimura, Tomohiro Terasaka, Nahoko Iwata, Takayuki Katsuyama, Motoshi Komatsubara, Ryota Nagao, Kenichi Inagaki, Fumio Otsuka

**Affiliations:** 1 Department of General Medicine, Okayama University Graduate School of Medicine, Dentistry and Pharmaceutical Sciences, 2-5-1 Shikata-cho, Kitaku, Okayama 700-8558, Japan; k_kimura14@yahoo.co.jp (K.K.); teras-t@cc.okayama-u.ac.jp (T.T.); nao53mayflower@gmail.com (N.I.); p4le6611@s.okayama-u.ac.jp (R.N.); 2 Department of Medicine and Clinical Science, Okayama University Graduate School of Medicine, Dentistry and Pharmaceutical Sciences, 2-5-1 Shikata-cho, Kitaku, Okayama 700-8558, Japan; takayukikatsuyama@gmail.com (T.K.); swe_etfish@yahoo.co.jp (M.K.); kenina@md.okayama-u.ac.jp (K.I.)

**Keywords:** androgen, bone morphogenetic protein (BMP), dihydrotestosteone (DHT), growth hormone (GH), osteoblasts

## Abstract

Osteoblasts undergo differentiation in response to various factors, including growth factors and steroids. Bone mass is diminished in androgen- and/or growth hormone (GH)-deficient patients. However the functional relationship between androgen and GH, and their combined effects on bone metabolism, remains unclear. Here we investigated the mutual effects of androgen and GH on osteoblastic marker expression using mouse myoblastic C2C12 and osteoblast-like MC3T3-E1 cells. Combined treatment with dihydrotestosterone (DHT) and GH enhanced BMP-2-induced expression of Runx2, ALP, and osteocalcin mRNA, compared with the individual treatments in C2C12 cells. Co-treatment with DHT and GH activated Smad1/5/8 phosphorylation, Id-1 transcription, and ALP activity induced by BMP-2 in C2C12 cells but not in MC3T3-E1 cells. The insulin-like growth factor (IGF-I) mRNA level was amplified by GH and BMP-2 treatment and was restored by co-treatment with DHT in C2C12 cells. The mRNA level of the IGF-I receptor was not significantly altered by GH or DHT, while it was increased by IGF-I. In addition, IGF-I treatment increased collagen-1 mRNA expression, whereas blockage of endogenous IGF-I activity using an anti-IGF-I antibody failed to suppress the effect of GH and DHT on BMP-2-induced Runx2 expression in C2C12 cells, suggesting that endogenous IGF-I was not substantially involved in the underlying GH actions. On the other hand, androgen receptor and GH receptor mRNA expression was suppressed by BMP-2 in both cell lines, implying the existence of a feedback action. Collectively the results showed that the combined effects of androgen and GH facilitated BMP-2-induced osteoblast differentiation at an early stage by upregulating BMP receptor signaling.

## 1. Introduction

Osteoblasts that have arisen from mesenchymal stem cell precursors undergo differentiation in response to various endocrine and autocrine/paracrine factors, including bone morphogenetic proteins (BMPs), growth hormone (GH), insulin-like growth factor-I (IGF-I), vitamin D, and steroids [1,2]. Among these, bone loss in patients with hypogonadism or hypopituitarism is closely related to decreases in sex steroids and growth hormone (GH). Estrogens are critical for the maintenance of bone mass in women, while a decrease in the androgen level also causes bone loss in men. Testosterone replacement is beneficial for the maintenance of bone mineral density (BMD) [3,4], in which both the effects of the androgen and estrogen converted from the androgen are likely to be involved [5].

On the other hand, GH also plays a key role in bone development and bone metabolism. It has been reported that bone mass is diminished in GH-deficient patients and that GH replacement is effective to reverse the bone loss. It has been shown that deficiencies of testosterone and GH are associated with low BMD and lead to an increase in fracture risk [6,7]. It has also been shown that testosterone replacement therapy and GH replacement therapy improve BMD in hypogonadal and GH-deficient male patients [8,9]. However the functional interaction of androgen and the GH/IGF-I axis in osteoblast differentiation and bone formation remains to be elucidated. It is also not clear whether androgen has an impact on bone metabolism via GH effects without IGF-I, through IGF-I action without GH, or independently of GH and IGF-I activity.

The pluripotent mesenchymal precursor cell line C2C12, a subclone of a mouse myoblastic cell line, and the osteoblast-like cell line MC3T3-E1 have been used as models to investigate the early and developed stages of osteoblast differentiation, respectively. Treatment with TGF-β ligands has distinct effects on differentiation of C2C12 cells, in which BMP ligands inhibit myoblast differentiation and facilitate osteoblastic differentiation [10]. Exposure to BMP ligands causes mesoderm induction and mesenchymal stem cell differentiation into chondrocytes and osteoblasts as well as differentiation of osteoprogenitor cells into osteoblasts. BMPs are also known to have roles in the regulation of embryological development and endocrine functions [11,12]. The biological functions of BMPs are mediated through the Smad signaling pathway via specific combinations of the corresponding BMP receptors. We previously reported mutual effects between BMP receptors and estrogen receptor (ER) signaling in the processes of osteoblast differentiation at the early stage of C2C12 cells [13,14] and bone mineralization at the later stage using MC3T3-E1 cells [15].

In the present study, we investigated the combined effects of androgen and GH on osteoblast marker expression in different stages of osteoblastic differentiation using two cell lines, with a focus on the interaction between BMP activity and pathways of the androgen receptor (AR) and the GH/IGF-I axis.

## 2. Materials and Methods

### 2.1. Reagents and Supplies

Dulbecco’s Modified Eagle’s Medium (DMEM), Minimum Essential Medium (MEM)-α, penicillin-streptomycin solution, and dimethylsulfoxide (DMSO) were purchased from Sigma-Aldrich Co., Ltd. (St. Louis, MO, USA). 5α-dihydrotestosterone (DHT), recombinant human somatotropin (GH), and IGF-I were also obtained from Sigma-Aldrich Co., Ltd. (St. Louis, MO, USA). Recombinant human BMP-2, anti-mouse IGF-I antibody, and normal goat IgG were purchased from R & D Systems Inc. (Minneapolis, MN, USA).

### 2.2. Cell Culture and Morphological Examination

C2C12 and MC3T3-E1 cell lines were obtained from American Type Culture Collection (Manassas, VA, USA). C2C12 cells and MC3T3-E1 cells were cultured in high-glucose DMEM and MEM-α, respectively, at 37 °C under a humid atmosphere of 95% air/5% CO_2_. Each medium was supplemented with 10% fetal calf serum (FCS) and penicillin-streptomycin solution. Changes in cell morphology were serially monitored using an inverted microscope.

### 2.3. RNA Extraction, RT-PCR, and Quantitative Real-Time PCR Analysis

To prepare total cellular RNA, cells were cultured in a 12-well plate (2 × 10^5^ viable cells/well) and treated with the indicated concentrations of BMP-2 in combination with DHT, GH, and IGF-I in serum-free DMEM. After 48-h culture, the medium was removed and the total cellular RNA was extracted using TRIzol^®^ (Invitrogen Corp., Carlsbad, CA, USA), quantified by measuring absorbance at 260 nm, and stored at −80 °C. The extracted RNA (1.0 μg) was subjected to a reverse transcription (RT) reaction using the First-Strand cDNA synthesis system^®^ (Invitrogen Corp.) with random hexamer (2 ng/μL), reverse transcriptase (200 U), and deoxynucleotide triphosphate (2.5 mM) at 42 °C for 55 min and at 70 °C for 10 min. PCR primer pairs, custom-ordered from Invitrogen Corp., were selected from different exons of the corresponding genes, with the primer pairs for osterix, alkaline phosphatase (ALP), runt-related transcription factor 2 (Runx2), osteocalcin, type-1 collagen (collagen-1), Id-1, GH receptor (GHR), IGF-I and IGF-I receptor (IGF-IR), and ribosomal protein L19 (RPL19) as a house-keeping gene being used as reported previously [13,14,15,16,17,18,19]. Primer pairs for the AR were 2521–2543 and 2711–2734 from GenBank accession #NM_013476. For the quantification of mRNA levels of target genes, real-time PCR was performed using the LightCycler^®^ Nano Real-Time PCR system (Roche Diagnostic Co., Tokyo, Japan) under optimized annealing conditions at 60–62 °C. The relative expression of each mRNA was calculated by the Δ threshold cycle (*Ct*) method, in which Δ*Ct* was the value obtained by subtracting the *Ct* value of RPL19 mRNA from the *Ct* value of the target mRNA, and the amount of target mRNA relative to RPL19 mRNA was expressed as 2^−(Δ*Ct*)^. The results were expressed as the ratio of target mRNA to RPL19 mRNA.

### 2.4. Western Immunoblot Analysis

C2C12 cells (1 × 10^5^ viable cells/well) were precultured in 12-well plates in DMEM containing 10% FCS for 24 h. After preculture, the medium was replaced with fresh serum-free medium, and the cells were treated with the indicated concentrations of DHT and GH for 48 h. Following stimulation with BMP-2 for 60 min, the cells were solubilized in 100 μL RIPA lysis buffer (Upstate Biotechnology, Inc., Lake Placid, NY, USA) containing 1 mM Na_3_VO_4_, 1 mM sodium fluoride, 2% sodium dodecyl sulfate, and 4% β-mercaptoethanol. The cell lysates were then subjected to SDS-PAGE/immunoblotting analysis using anti-phospho-Smad1/5/8 (pSmad1/5/8) and total-Smad1 (tSmad1) (Cell Signaling Technology, Inc., Beverly, MA, USA). The relative integrated density of each protein band was digitized and analyzed by the C-DiGit^®^ Blot Scanner System (LI-COR Biosciences, Lincoln, NE, USA). For evaluating pSmad1/5/8 levels, ratios of the signal intensities of pSmad/tSmad1 were calculated.

### 2.5. ALP Activity Assay

C2C12 and MC3T3-E1 cells were cultured in a 12-well plate (1 × 10^5^ viable cells/well) and treated with the indicated concentrations of BMP-2, DHT and GH. After 72-h culture, cells were washed with PBS and lysed with Cell Culture Lysis Reagent (Toyobo, Osaka, Japan), and then cellular ALP activity in the lystes was determined by the fluorometric assay for 4-nitrophenol (Wako Chemical Co., Osaka, Japan), as we previously reported [15,19].

### 2.6. Statistical Analysis

Results are shown as means ± SEM of data from at least three separate experiments, each performed with triplicate samples. Differences between groups were analyzed for statistical significance using ANOVA with Fisher’s PLSD test or the unpaired *t*-test, when appropriate, to determine differences (StatView 5.0 software, Abacus Concepts, Inc., Berkeley, CA, USA). *p* values < 0.05 were accepted as statistically significant.

## 3. Results

**Figure 1:**
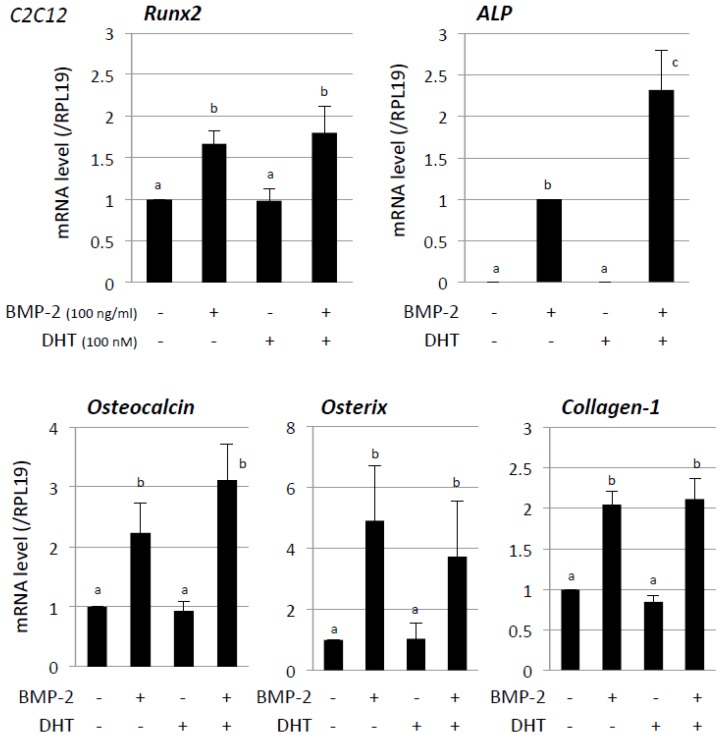
Effects of androgen on BMP-induced osteoblast marker expression. C2C12 cells (2 × 10^5^ cells/well) were treated with the indicated concentrations of DHT, GH, and BMP-2 in serum-free DMEM. After 48-h culture, total cellular RNA was extracted and mRNA levels of Runx2, ALP, osteocalcin, osterix, collagen-1, and Id-1 were examined by real-time RT-PCR. The expression levels of target genes were standardized by RPL19 mRNA levels in each sample. Results in all panels are shown as means ± SEM of data from at least three separate experiments, each performed with triplicate samples. The results were analyzed by ANOVA. For each result within a panel, the values with different superscript letters are significantly different at *p* < 0.05.

First the individual effects of androgen on BMP-2-induced osteoblast differentiation were examined using C2C12 cells (Figure 1). Treatment with BMP-2 (100 ng/mL) for 48 h significantly increased mRNA levels of osteoblast differentiation markers, including Runx2, ALP, osteocalcin, osterix and collagen-1, in C2C12 cells. It has been reported that 10^−7^ M of DHT, within a range of 10^−6^ to 10^−9^ M, stably induces myogenic marker and myotube protein expression in C2C12 cells [20,21,22]. Based on our pilot study using 10^−8^ to 10^−6^ M of DHT, a pharmacological dose, 10^−7^ M of DHT, was the minimal dose enabling amplification of BMP-induced signaling in C2C12 cells and that dose was selected for the following study. As shown in Figure 1, DHT alone (100 nM) had no effect on mRNA levels of these osteoblastic markers. It was found that DHT treatment (100 nM) significantly increased BMP-2 (100 ng/mL)-induced ALP mRNA levels, whereas BMP-2-induced expression of Runx2, osteocalcin, osterix, and collagen-1 was not significantly affected by DHT treatment (Figure 1).

**Figure 2:**
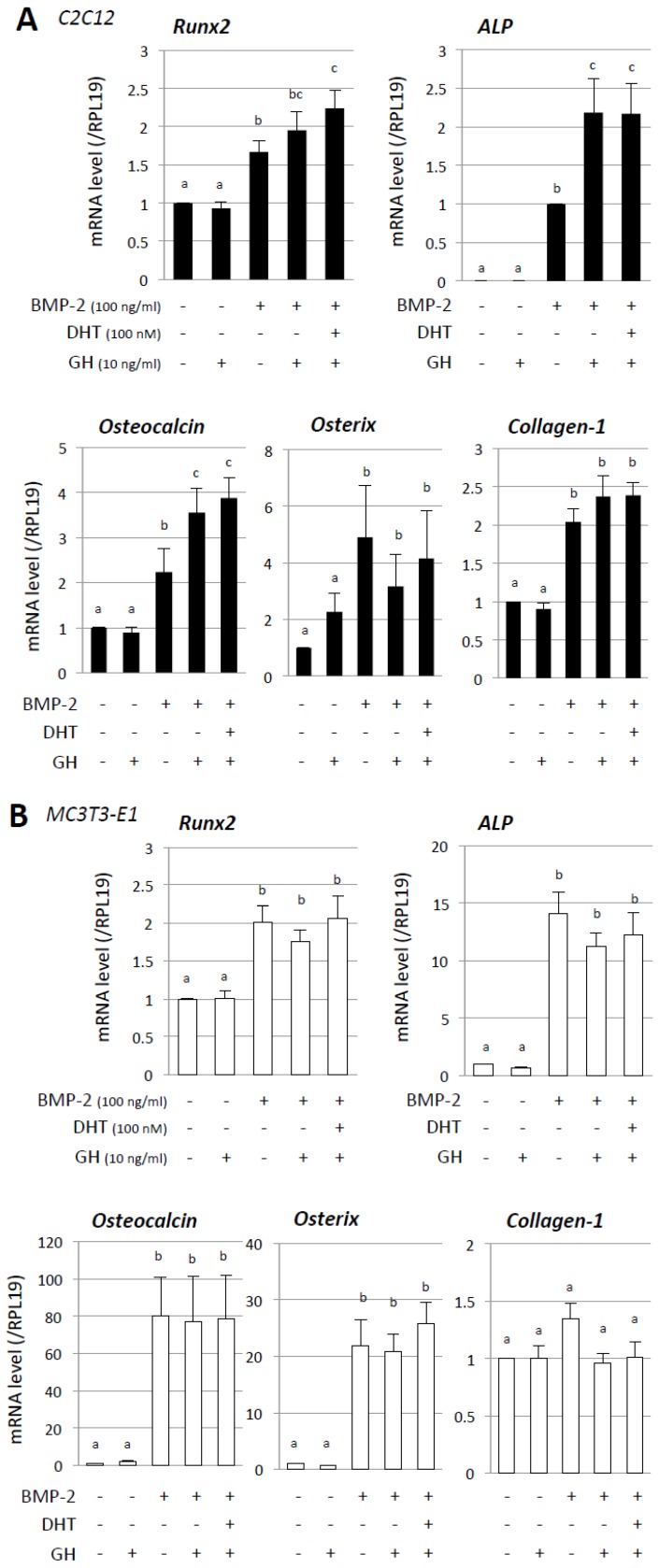
Combined effects of androgen and GH on BMP-induced osteoblast marker expression. (**A**) C2C12 and (**B**) MC3T3-E1 cells (2 × 10^5^ cells/well) were treated with the indicated concentrations of DHT, GH, and BMP-2 in a serum-free medium. After 48-h culture, total cellular RNA was extracted and mRNA levels of Id-1 were examined by real-time RT-PCR. The expression levels of target genes were standardized by RPL19 mRNA levels in each sample. Results in all panels are shown as means ± SEM of data from at least three separate experiments, each performed with triplicate samples. The results were analyzed by ANOVA. For each result within a panel, the values with different superscript letters are significantly different at *p* < 0.05.

We next investigated the combined effects of androgen and GH on BMP-2-induced expression of osteoblast differentiation markers (Figure 2). The concentration of GH was selected on the basis of lower but stable concentrations that can induce endogenous IGF-I expression. It has been reported that stimulation of IGF-I mRNA expression occurs at doses of GH higher than 7.5 ng/mL [23] and that GH at a concentration of about 10 ng/mL can activate the key intracellular signaling protein Stat5 in C2C12 cells [24]. As shown in Figure 2A, treatment with GH increased mRNA expression of ALP and osteocalcin induced by BMP-2 (100 ng/mL) in C2C12 cells. Furthermore combined treatment with DHT (100 nM) and GH (10 ng/mL) significantly increased mRNA expression of Runx2, ALP, and osteocalcin induced by BMP-2 (100 ng/mL) in C2C12 cells (Figure 2A). In contrast, the BMP-2-induced mRNA levels of osterix and collagen-1 were not significantly altered by co-treatment with DHT and GH. In the case of MC3T3-E1 cells, co-treatment with DHT (100 nM) and GH (10 ng/mL) did not affect the mRNA expression of Runx2, ALP, osteocalcin, or osterix induced by BMP-2 (100 ng/mL) (Figure 2B). In addition, collagen-1 mRNA was not significantly induced by BMP-2 in MC3T3-E1 cells. Thus it was found that combined treatment with androgen and GH enabled enhancement of BMP-2-induced osteoblast differentiation at the early phase as shown in C2C12 cells.

**Figure 3:**
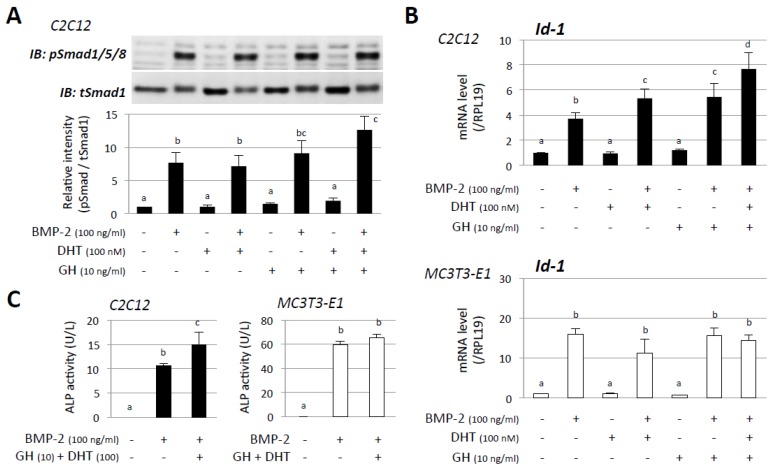
Effects of androgen and GH on BMP-receptor signal activation. (**A**) C2C12 cells (1 × 10^5^ cells/well) were precultured in a serum-free condition in the absence or presence of DHT and GH for 48 h and were then stimulated with BMP-2. After 1-h culture with BMP-2 treatment, cells were lysed and subjected to SDS-PAGE/immunoblotting (IB) analysis using antibodies that can detect pSmad1/5/8 and tSmad1. The integrated signal density of each protein band was digitally analyzed, and the ratios of signal intensities of pSmad/tSmad1 were calculated; (**B**) C2C12 and MC3T3-E1 cells (2 × 10^5^ cells/well) were treated with the indicated concentrations of DHT, GH, and BMP-2 in a serum-free medium. After 48-h culture, total cellular RNA was extracted and mRNA levels of Id-1 were examined by real-time RT-PCR. The expression levels of the target gene were standardized by RPL19 mRNA levels in each sample; (**C**) C2C12 and MC3T3-E1 cells (1 × 10^5^ cells/well) were treated with the indicated concentrations of BMP-2, DHT, and GH in a serum-free medium for 72 h, and then ALP activity in the cell lysates was determined. Results in all panels are shown as means ± SEM of data from at least three separate experiments, each performed with triplicate samples. The results were analyzed by ANOVA. For each result within a panel, the values with different superscript letters are significantly different at *p* < 0.05.

To clarify the combined effects of androgen and GH on BMP-receptor signaling, changes in Smad1/5/8 phosphorylation and Id-1 mRNA expression were evaluated. As shown in Figure 3A, stimulation with BMP-2 (100 ng/mL) readily activated Smad1/5/8 phosphorylation in C2C12 cells for 60 min, whereas DHT (100 nM) or GH (100 ng/mL) had no effect on Smad1/5/8 phosphorylation. It was notable that BMP-2-induced phosphorylation of Smad1/5/8 was significantly enhanced by combined treatment with DHT and GH for 60 min (Figure 3A), although individual treatment with DHT or GH was not adequate to enhance BMP-2-induced Smad phosphorylation. A precise quantification, by measuring BMP target gene Id-1 mRNA levels, showed that treatment with DHT (100 nM) or GH (10 ng/mL) and, of note, combined treatment with DHT and GH significantly enhanced the levels of Id-1 mRNA expression induced by BMP-2 treatment (100 ng/mL) for 48 h in C2C12 cells (Figure 3B, *upper panel*). In contrast, the enhancing effect of DHT and GH on BMP-induced Id-1 expression was not observed in MC3T3-E1 cells (Figure 3B, *lower panel*). To confirm the combined effects of DHT (100 nM) and GH (10 ng/mL) on osteoblastic activity induced by BMP-2 (100 ng/mL), ALP activity was evaluated in cell lysates extracted from these cells (Figure 3C). In accordance with the results for Smad phosphorylation and Id-1 mRNA level, it was shown that combined effects of DHT and GH increased BMP-2-induced cellular ALP activity in C2C12 cells but not in MC3T3 cells.

**Figure 4:**
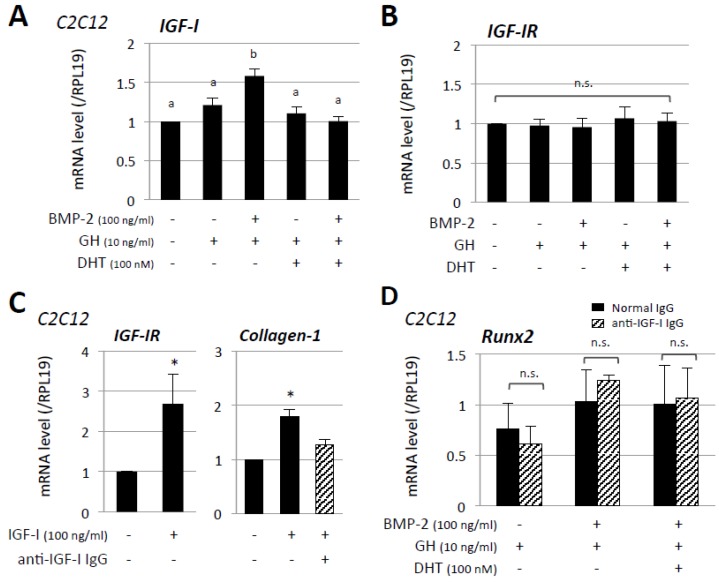
Involvement of IGF-I in osteoblast marker expression induced by BMP-2, androgen, and GH. (**A**,**B**) C2C12 and MC3T3-E1 cells (2 × 10^5^ cells/well) were treated with the indicated concentrations of DHT, GH, and BMP-2 in a serum-free medium. After 48-h culture, total cellular RNA was extracted and mRNA levels of IGF-I and IGF-IR were examined by real-time RT-PCR. The expression levels of target genes were standardized by RPL19 mRNA levels in each sample; (**C**) After C2C12 cells (2 × 10^5^ cells/well) had been treated with the indicated concentrations of IGF-I and anti-IGF-I IgG in a serum-free condition for 48 h, total cellular RNA was extracted and mRNA levels of IGF-IR and collagen-1 were examined by real-time RT-PCR. The expression levels of target genes were standardized by RPL19 mRNA levels in each sample; (**D**) BMP-2, DHT and GH were added to the cell culture in combination with either normal or anti-IGF-I IgG (1 μg/mL) in a serum-free condition for 48 h. The expression levels of Runx2 were determined by real-time RT-PCR and the expression levels of Runx2 mRNA were standardized by RPL19 mRNA levels in each sample. Results in all panels are shown as means ± SEM of data from at least three separate experiments, each performed with triplicate samples. The results were analyzed by ANOVA and the unpaired *t*-test. For each result within a panel, the values with different superscript letters are significantly different at *p* < 0.05 (**A**,**B**). * *p* < 0.05 vs. control group (**C**). n.s., not significant.

Of interest, as shown in Figure 4A, the IGF-I mRNA level was significantly increased by co-treatment with GH (10 ng/mL) and BMP-2 (100 ng/mL) in C2C12 cells. However the enhancement of IGF-I mRNA was restored in the presence of DHT (100 nM), suggesting that androgen counteracts GH action or GHR signaling. In addition, the IGF-IR mRNA level was not significantly affected by treatment with BMP-2, DHT, or GH (Figure 4B). To determine the cellular responsiveness to exogenous IGF-I, C2C12 cells were treated with IGF-I. As shown in Figure 4C, treatment with IGF-I (100 ng/mL) for 48 h resulted in significant increases in mRNA levels of IGF-IR and collagen-1, which were previously reported in human tissues and rat smooth muscle cells [25,26].

IGF-I-induced enhancement of collagen-1 mRNA was restored in the presence of anti-IGF-I IgG (Figure 4C), suggesting neutralization of IGF-I activity by treatment with anti-IGF-I IgG. Furthermore blockage of endogenous IGF-I activity by using either anti-IGF-I IgG or normal IgG (1 μg/mL) failed to suppress the effects of GH or combined treatment with DHT and GH on BMP-2-induced Runx2 mRNA expression (Figure 4D), indicating that endogenous IGF-I was not likely to be involved in the direct enhancement of GH actions in the presence of BMP-2 and androgen.

**Figure 5:**
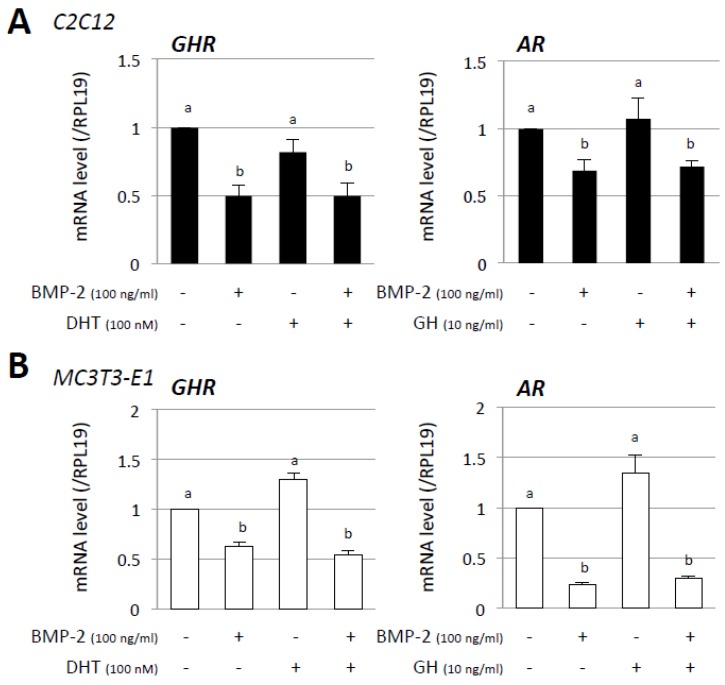
Mutual effects of androgen and GH on BMP-induced osteoblast marker expression. (**A**) C2C12 and (**B**) MC3T3-E1 cells (2 × 10^5^ cells/well) were treated with the indicated concentrations of DHT, GH, and BMP-2 in a serum-free medium. After 48-h culture, total cellular RNA was extracted and mRNA levels of GHR and AR were examined by real-time RT-PCR. The expression levels of target genes were standardized by RPL19 mRNA levels in each sample. Results in all panels are shown as means ± SEM of data from at least three separate experiments, each performed with triplicate samples. The results were analyzed by ANOVA. For each result within a panel, the values with different superscript letters are significantly different at *p* < 0.05.

To investigate the mutual effects of androgen and GH on BMP-2-induced osteoblast differentiation, changes in the expression of AR and GHR were examined. As shown in Figure 5, GHR mRNA expression was suppressed by treatment with BMP-2 (100 ng/mL), regardless of the presence of DHT (100 nM), whereas DHT (100 nM) did not affect GHR expression in either C2C12 cells (Figure 5A) or MC3T3-E1 cells (Figure 5B). AR mRNA expression was also suppressed by BMP-2 (100 ng/mL) regardless of the presence of GH (10 ng/mL), while GH (10 ng/mL) did not affect AR expression in either C2C12 cells (Figure 5A) or MC3T3-E1 cells (Figure 5B).

## 4. Discussion

**Figure 6:**
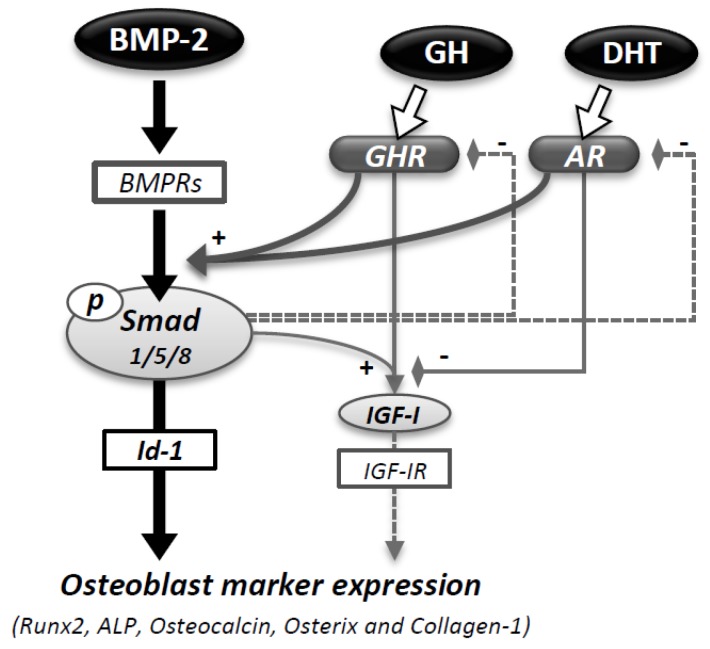
Interaction of androgen and GH in BMP-induced osteoblast marker expression. Combined treatment with DHT and GH enhanced BMP-2-induced expression of osteoblast markers. DHT and GH augmented BMP-2-induced Smad1/5/8 signaling and Id-1 transcription in C2C12 cells. The IGF-I mRNA level was amplified by GH and BMP-2, and the effect was reversed by DHT. The expression of AR and GHR was suppressed by BMP-2, suggesting a possible feedback loop.

In the present study, it was found that combined treatment with androgen and GH significantly increased the levels of Runx2, ALP, and osteocalcin mRNA induced by BMP-2, although the effect of androgen alone was less effective for the osteoblast marker expression in C2C12 cells. It was also revealed that combined treatment with DHT and GH augmented BMP-2-induced activity for Smad1/5/8 signaling, Id-1 transcription, and ALP activity in C2C12 cells but not in MC3T3-E1 cells, suggesting the significance of the mutual activity of androgen and the GH/IGF-I axis, predominantly in the early phase of osteoblastic differentiation (Figure 6).

Replacement of testosterone for male patients with androgen deficiency improves their BMD and bone architecture [9]. These changes are mediated, at least in part, by conversion of testosterone to estradiol [5]. In the present study, we therefore utilized DHT, instead of testosterone, as an androgen in order to assess the pure AR action without estrogen converted from testosterone. GH replacement for male cases with GH deficiency has also been reported to improve BMD [8,27]. In animal studies, male mice lacking GHR specifically in osteoblasts had a dramatic skeletal-feminization phenotype of the femur, while female mice lacking the specified GHR had a significantly reduced number of osteoblasts [28]. It has also been shown that testosterone and GH act synergistically to induce protein synthesis and elicit anabolic effects [29,30], while it has also been reported that combined treatment for males with hypopituitarism did not improve parameters of the distal tibia more than treatment with testosterone alone [31]. This discrepancy may imply that androgen aromatization is rather important for explaining the combined effects. However the mutual effects of androgen and GH on the regulation of bone metabolism have yet to be fully explored.

A lack of apparent additive effects of GH on androgen action in a clinical study [31] might be associated with the cellular changes in IGF-I, GHR, and AR expression in the process of osteoblast differentiation. In the present study, the IGF-I mRNA level was amplified by GH treatment in the presence of BMP-2; however the addition of DHT decreased the IGF-I expression induced by GH and BMP-2 in C2C12 cells (Figure 6). The expression levels of GHR and AR were also modulated by co-existing BMP-2 action. The mRNA level of IGF-IR was not significantly altered by GH treatment, while it was increased by IGF-I treatment. The neutralization of endogenous IGF-I activity using an anti-IGF-I antibody failed to suppress the combined effects of DHT and GH on BMP-2-induced osteoblastic differentiation, implying that endogenous IGF-I was not potentially involved in the mutual actions of androgen and GH (Figure 6).

These results seem to be consistent with the changes of skeletal structure shown in IGF-I-deficient mice [32], in which the bones were smaller than those of wild type. Nevertheless the periosteal bone formation and trabecular architecture were maintained for size [32], suggesting that IGF-I may not be substantially required for the formation of developed bone. It is possible that GH and IGF-I have functionally independent activities depending on the stage of bone growth [33]. The finding that replacement of GH increases bone formation in IGF-I knockout mice [32] also suggests independent roles for GH and IGF-I in bone formation.

The roles of the GH/IGF-I axis of bone growth in males and females are still unclear. Regarding the functional link between GH action and AR, it was revealed that BMP-2 has suppressive effects on GHR and AR expression in both C2C12 and MC3T3-E1 cells. The suppressive effects of BMP-2 on AR and GHR expression in both cell lines may imply the existence of a feedback property in the process of osteoblast differentiation (Figure 6). In the present study, combined treatment with DHT and GH facilitated BMP-2-induced osteoblast marker expression by upregulating the Smad1/5/8 or Id-1 signaling pathways in C2C12 cells, though the mutual interfering effects of androgen, GH, and BMP-2 should also be considered in this situation. In this regard, Klover et al. [34] reported the importance of the transcription factor STAT5a/b in skeletal muscle. Using muscle-specific STAT5-null mice and C2C12 cells, they demonstrated that the expression of AR is regulated by GH action through STAT5 signaling. On the other hand, IGF-I mRNA induction by co-treatment with GH and BMP-2 was abolished by treatment with DHT in our experiments, implying that androgen also counteracts GH action or GHR signaling. The suppressor of cytokine signaling 2, as a mediator of crosstalk between androgen and GH, might be functionally involved in this mechanism [35]. Venken et al. also showed that DHT administration in GHR-null and orchidectomized male rats restored bone modeling, suggesting that DHT has an independent effect on bone metabolism [36]. Further study is needed to clarify the mutual regulation among the activities of AR, GHR, IGF-IR, and IGF binding proteins (IGFBPs) in the process of osteoblast differentiation in various growth stages.

Collectively the results of this study showed that combined treatment with androgen and GH enabled enhancement of osteoblast marker expression induced by BMP-2 at the early phase, as demonstrated in C2C12 cells compared with MC3T3-E1 cells (Figure 6). BMP-2 also elicited regulatory effects on androgen and GH activities and androgen impaired IGF-I expression induced by GH and BMP-2. These findings suggested that the combined activities of androgen and GH have roles in facilitating BMP-2-induced osteoblast differentiation by upregulating Smad signaling but, inversely, the antagonistic and/or feedback activities of androgen and GH come into existence (Figure 6). Taken together, this mutual regulation of androgen and GH/IGF-I signaling might be functionally linked to the process of osteoblastic differentiation shown in the clinical setting of the combined replacement of androgen and GH.

## Abbreviations

AR	androgen receptor
BMD	bone mineral density
BMP	bone morphogenetic protein
DHT	dihydrotestosterone
GH	growth hormone
GHR	GH receptor
IGFBP	IGF binding protein
IGF-I	Insulin-like growth factor-I
IGF-IR	IGF-I receptor

## References

[1] Spelsberg T.C., Subramaniam M., Riggs B.L., Khosla S. (1999). The actions and interactions of sex steroids and growth factors/cytokines on the skeleton. Mol. Endocrinol..

[2] Gurlek A., Pittelkow M.R., Kumar R. (2002). Modulation of growth factor/cytokine synthesis and signaling by 1α,25-dihydroxyvitamin D_3_: Implications in cell growth and differentiation. Endocr. Rev..

[3] Sinnesael M., Claessens F., Boonen S., Vanderschueren D. (2013). Novel insights in the regulation and mechanism of androgen action on bone. Curr. Opin. Endocrinol. Diabetes Obes..

[4] Cauley J.A. (2015). Estrogen and bone health in men and women. Steroids.

[5] Khosla S., Melton L.J., Riggs B.L. (2002). Clinical review 144: Estrogen and the male skeleton. J. Clin. Endocrinol. Metab..

[6] Tritos N.A., Greenspan S.L., King D., Hamrahian A., Cook D.M., Jonsson P.J., Wajnrajch M.P., Koltowska-Haggstrom M., Biller B.M. (2011). Unreplaced sex steroid deficiency, corticotropin deficiency, and lower IGF-I are associated with lower bone mineral density in adults with growth hormone deficiency: A KIMS database analysis. J. Clin. Endocrinol. Metab..

[7] Wuster C., Abs R., Bengtsson B.A., Bennmarker H., Feldt-Rasmussen U., Hernberg-Stahl E., Monson J.P., Westberg B., Wilton P., Group K.S. (2001). The influence of growth hormone deficiency, growth hormone replacement therapy, and other aspects of hypopituitarism on fracture rate and bone mineral density. J. Bone Miner. Res..

[8] Snyder P.J., Biller B.M., Zagar A., Jackson I., Arafah B.M., Nippoldt T.B., Cook D.M., Mooradian A.D., Kwan A., Scism-Bacon J. (2007). Effect of growth hormone replacement on BMD in adult-onset growth hormone deficiency. J. Bone Miner. Res..

[9] Snyder P.J., Peachey H., Berlin J.A., Hannoush P., Haddad G., Dlewati A., Santanna J., Loh L., Lenrow D.A., Holmes J.H. (2000). Effects of testosterone replacement in hypogonadal men. J. Clin. Endocrinol. Metab..

[10] Katagiri T., Yamaguchi A., Komaki M., Abe E., Takahashi N., Ikeda T., Rosen V., Wozney J.M., Fujisawa-Sehara A., Suda T. (1994). Bone morphogenetic protein-2 converts the differentiation pathway of C2C12 myoblasts into the osteoblast lineage. J. Cell Biol..

[11] Shimasaki S., Moore R.K., Otsuka F., Erickson G.F. (2004). The bone morphogenetic protein system in mammalian reproduction. Endocr. Rev..

[12] Otsuka F. (2010). Multiple endocrine regulation by bone morphogenetic protein system. Endocr. J..

[13] Matsumoto Y., Otsuka F., Takano M., Mukai T., Yamanaka R., Takeda M., Miyoshi T., Inagaki K., Sada K.E., Makino H. (2010). Estrogen and glucocorticoid regulate osteoblast differentiation through the interaction of bone morphogenetic protein-2 and tumor necrosis factor-α in C2C12 cells. Mol. Cell Endocrinol..

[14] Takano M., Otsuka F., Matsumoto Y., Inagaki K., Takeda M., Nakamura E., Tsukamoto N., Miyoshi T., Sada K.E., Makino H. (2012). Peroxisome proliferator-activated receptor activity is involved in the osteoblastic differentiation regulated by bone morphogenetic proteins and tumor necrosis factor-α. Mol. Cell Endocrinol..

[15] Matsumoto Y., Otsuka F., Takano-Narazaki M., Katsuyama T., Nakamura E., Tsukamoto N., Inagaki K., Sada K.E., Makino H. (2013). Estrogen facilitates osteoblast differentiation by upregulating bone morphogenetic protein-4 signaling. Steroids.

[16] Mukai T., Otsuka F., Otani H., Yamashita M., Takasugi K., Inagaki K., Yamamura M., Makino H. (2007). TNF-α inhibits BMP-induced osteoblast differentiation through activating SAPK/JNK signaling. Biochem. Biophys. Res. Commun..

[17] Nakamura E., Otsuka F., Inagaki K., Miyoshi T., Matsumoto Y., Ogura K., Tsukamoto N., Takeda M., Makino H. (2012). Mutual regulation of growth hormone and bone morphogenetic protein system in steroidogenesis by rat granulosa cells. Endocrinology.

[18] Katsuyama T., Otsuka F., Terasaka T., Inagaki K., Takano-Narazaki M., Matsumoto Y., Sada K.E., Makino H. (2015). Regulatory effects of fibroblast growth factor-8 and tumor necrosis factor-α on osteoblast marker expression induced by bone morphogenetic protein-2. Peptides.

[19] Yamashita M., Otsuka F., Mukai T., Otani H., Inagaki K., Miyoshi T., Goto J., Yamamura M., Makino H. (2008). Simvastatin antagonizes tumor necrosis factor-α inhibition of bone morphogenetic proteins-2-induced osteoblast differentiation by regulating Smad signaling and Ras/Rho-mitogen-activated protein kinase pathway. J. Endocrinol..

[20] Diel P., Baadners D., Schlupmann K., Velders M., Schwarz J.P. (2008). C2C12 myoblastoma cell differentiation and proliferation is stimulated by androgens and associated with a modulation of myostatin and Pax7 expression. J. Mol. Endocrinol..

[21] Kanno Y., Ota R., Someya K., Kusakabe T., Kato K., Inouye Y. (2013). Selective androgen receptor modulator, YK11, regulates myogenic differentiation of C2C12 myoblasts by follistatin expression. Biol. Pharm. Bull..

[22] Chen Y., Lee N.K., Zajac J.D., MacLean H.E. (2008). Generation and analysis of an androgen-responsive myoblast cell line indicates that androgens regulate myotube protein accretion. J. Endocrinol. Investig..

[23] Frost R.A., Nystrom G.J., Lang C.H. (2002). Regulation of IGF-I mRNA and signal transducers and activators of transcription-3 and -5 (Stat-3 and -5) by GH in C2C12 myoblasts. Endocrinology.

[24] Sadowski C.L., Wheeler T.T., Wang L.H., Sadowski H.B. (2001). GH regulation of IGF-I and suppressor of cytokine signaling gene expression in C2C12 skeletal muscle cells. Endocrinology.

[25] Hansen M., Boesen A., Holm L., Flyvbjerg A., Langberg H., Kjaer M. (2013). Local administration of insulin-like growth factor-I (IGF-I) stimulates tendon collagen synthesis in humans. Scand. J. Med. Sci. Sports.

[26] Xin X., Hou Y.T., Li L., Schmiedlin-Ren P., Christman G.M., Cheng H.L., Bitar K.N., Zimmermann E.M. (2004). IGF-I increases IGFBP-5 and collagen α_1_(I) mRNAs by the MAPK pathway in rat intestinal smooth muscle cells. Am. J. Physiol. Gastrointest. Liver Physiol..

[27] Bex M., Abs R., Maiter D., Beckers A., Lamberigts G., Bouillon R. (2002). The effects of growth hormone replacement therapy on bone metabolism in adult-onset growth hormone deficiency: A 2-year open randomized controlled multicenter trial. J. Bone Miner. Res..

[28] Singhal V., Goh B.C., Bouxsein M.L., Faugere M.C., DiGirolamo D.J. (2013). Osteoblast-restricted Disruption of the Growth Hormone Receptor in Mice Results in Sexually Dimorphic Skeletal Phenotypes. Bone Res..

[29] Blackman M.R., Sorkin J.D., Munzer T., Bellantoni M.F., Busby-Whitehead J., Stevens T.E., Jayme J., O'Connor K.G., Christmas C., Tobin J.D. (2002). Growth hormone and sex steroid administration in healthy aged women and men: A randomized controlled trial. JAMA.

[30] Mauras N., Rini A., Welch S., Sager B., Murphy S.P. (2003). Synergistic effects of testosterone and growth hormone on protein metabolism and body composition in prepubertal boys. Metabolism.

[31] Al Mukaddam M., Rajapakse C.S., Bhagat Y.A., Wehrli F.W., Guo W., Peachey H., LeBeau S.O., Zemel B.S., Wang C., Swerdloff R.S. (2014). Effects of testosterone and growth hormone on the structural and mechanical properties of bone by micro-MRI in the distal tibia of men with hypopituitarism. J. Clin. Endocrinol. Metab..

[32] Bikle D., Majumdar S., Laib A., Powell-Braxton L., Rosen C., Beamer W., Nauman E., Leary C., Halloran B. (2001). The skeletal structure of insulin-like growth factor I-deficient mice. J. Bone Miner. Res..

[33] Olson L.E., Ohlsson C., Mohan S. (2011). The role of GH/IGF-I-mediated mechanisms in sex differences in cortical bone size in mice. Calcif. Tissue Int..

[34] Klover P., Chen W., Zhu B.M., Hennighausen L. (2009). Skeletal muscle growth and fiber composition in mice are regulated through the transcription factors STAT5a/b: Linking growth hormone to the androgen receptor. FASEB J..

[35] Iglesias-Gato D., Chuan Y.C., Wikstrom P., Augsten S., Jiang N., Niu Y., Seipel A., Danneman D., Vermeij M., Fernandez-Perez L. (2014). SOCS_2_ mediates the cross talk between androgen and growth hormone signaling in prostate cancer. Carcinogenesis.

[36] Venken K., Moverare-Skrtic S., Kopchick J.J., Coschigano K.T., Ohlsson C., Boonen S., Bouillon R., Vanderschueren D. (2007). Impact of androgens, growth hormone, and IGF-I on bone and muscle in male mice during puberty. J. Bone Miner. Res..

